# MCDHGN: heterogeneous network-based cancer driver gene prediction and interpretability analysis

**DOI:** 10.1093/bioinformatics/btae362

**Published:** 2024-06-12

**Authors:** Lexiang Wang, Jingli Zhou, Xuan Wang, Yadong Wang, Junyi Li

**Affiliations:** School of Computer Science and Technology, Harbin Institute of Technology (Shenzhen), Shenzhen 518055, China; School of Computer Science and Technology, Harbin Institute of Technology (Shenzhen), Shenzhen 518055, China; School of Computer Science and Technology, Harbin Institute of Technology (Shenzhen), Shenzhen 518055, China; Guangdong Provincial Key Laboratory of Novel Security Intelligence Technologies, Harbin Institute of Technology (Shenzhen), Shenzhen 518055, China; Center for Bioinformatics, Faculty of Computing, Harbin Institute of Technology, Harbin, Heilongjiang 150001, China; Ministry of Education, Key Laboratory of Biological Bigdata, Harbin Institute of Technology, Harbin, Heilongjiang 150001, China; School of Computer Science and Technology, Harbin Institute of Technology (Shenzhen), Shenzhen 518055, China; Guangdong Provincial Key Laboratory of Novel Security Intelligence Technologies, Harbin Institute of Technology (Shenzhen), Shenzhen 518055, China; Ministry of Education, Key Laboratory of Biological Bigdata, Harbin Institute of Technology, Harbin, Heilongjiang 150001, China

## Abstract

**Motivation:**

Accurately predicting the driver genes of cancer is of great significance for carcinogenesis progress research and cancer treatment. In recent years, more and more deep-learning-based methods have been used for predicting cancer driver genes. However, deep-learning algorithms often have black box properties and cannot interpret the output results. Here, we propose a novel cancer driver gene mining method based on heterogeneous network meta-paths (MCDHGN), which uses meta-path aggregation to enhance the interpretability of predictions.

**Results:**

MCDHGN constructs a heterogeneous network by using several types of multi-omics data that are biologically linked to genes. And the differential probabilities of SNV, DNA methylation, and gene expression data between cancerous tissues and normal tissues are extracted as initial features of genes. Nine meta-paths are manually selected, and the representation vectors obtained by aggregating information within and across meta-path nodes are used as new features for subsequent classification and prediction tasks. By comparing with eight homogeneous and heterogeneous network models on two pan-cancer datasets, MCDHGN has better performance on AUC and AUPR values. Additionally, MCDHGN provides interpretability of predicted cancer driver genes through the varying weights of biologically meaningful meta-paths.

**Availability and implementation:**

https://github.com/1160300611/MCDHGN

## 1 Introduction

Mutations occurring in somatic cells that confer a preferential growth advantage to tumor cells are termed driver mutations. The genes responsible for driving these mutations are referred to as driver genes. The overarching objective of the International Cancer Genome Consortium has consistently revolved around comprehending, elucidating, and pinpointing cancer driver genes. But finding cancer driver genes through traditional medical approaches requires significant time and research costs. With bioinformatics and high-throughput databases, we can accelerate this process through computational biology. Conventional machine learning approaches concentrate on genomic sequence data and identify cancer driver genes by evaluating gene sequence mutation probabilities, hotspots, and frequencies (Han *et al.* 2019), such as the MutsigCV method proposed by Lawrence *et al.* (2013). Dietlein *et al.* (2020) proposed a comprehensive oncogenomics workflow for constructing a compilation of cancer driver genes. This workflow integrates several advanced positive selection signal bias techniques and outlines three fundamental steps for handling cancer genomics data: preprocessing, implementing seven complementary driver gene identification methods, and combining candidate driver genes identified by each method through weighted voting. The TCGA (Tomczak *et al.* 2015) initiative has introduced a pan-cancer network database incorporating multi-omic data (Gibbons and Creighton 2018), standardizing expression within the field of cancer research. This initiative increases data volume and standardizes data formats, thereby facilitating the application of deep learning in predicting cancer driver genes. Elmarakeby *et al.* (2021) aggregated initial information from cases by building a feature aggregation architecture that is rich in biological information and conforms to a biological hierarchical system, and clinically validated it in the field of prostate cancer. Their work is more in line with clinical medicine’s need for explainability of cancer drug resistance. Compared with traditional network analysis, graph neural networks can obtain a variable number of neighbor node feature values and a local topology composed of neighbor nodes that are important to the classification result, and thus better perform the classification task of the nodes. Schulte-Sasse *et al.* (2021) introduced the EMOGI algorithm in their publication, which uses multi-omics data as node features, and uses graph neural network algorithms in a semi-supervised manner for training in protein interactions networks to learn the complex non-linear structures to identify the oncogenes and non-oncogenes. Building on the GCN method and homomorphic networks, Peng *et al.* (2022) made further extensions. Node classification is used as the primary task, with link prediction between pairs of nodes as a secondary task. These two tasks share two Chebyshev GCN layers and optimize two different objective functions. Zhao *et al.* (2022; MODIG) further enriched and expanded the gene network by constructing a multi-dimensional homogenous network of genes using five types of direct and indirect gene relationships. It obtains the representation vectors of gene nodes through the aggregated representations across these five networks. The MTGCL method (Peng *et al.* 2024) constructs three different gene network views from multi-omics data, essentially forming a multi-dimensional homogeneous network. This approach applies graph contrastive learning with shared parameters across these networks to learn consistent gene features across networks.

Existing network-based studies often involve only one type of node, blurring the labels of other molecular nodes or physiological roles that enable the two gene nodes to interact. This limitation hinders the interpretability of biological analyses. This study aims to make better use of biological high-throughput data and the interaction between genomics data and other histological data, and tightly integrate biological a priori knowledge in order to improve the predictive accuracy of cancer driver genes and the interpretability of the results. We constructed a heterogeneous network containing multi-omics data, nodes of multiple biological entity types and multiple interrelationships, and applied advanced heterogeneous network characterization algorithms to the characterization mining of cancer-related genes, with the main task of classifying and predicting cancer driver genes in the biological network. At the same time, we also pursue to combine the weights contributed by the meta-paths neighbor in the network to give some explanations for the cancer driver genes predicted by our model. Make the result to be more relevant to biological analysis, and to be more acceptable to the biological community.

## 2 Materials and methods

### 2.1 Datasets

Some cancer-driving genes not only affect one type of cancer but play a role in the development of various cancer types and subtypes (ICGC/TCGA Pan-Cancer Analysis of Whole Genomes Consortium 2020). Furthermore, in network-based studies, our main task is described as the classification of gene nodes. In this semi-supervised learning task, the quantity and quality of node labels significantly impact the classification outcomes. If we only use labels for driver genes related to a specific type of cancer, the number of positively labeled nodes is too small relative to the entire gene collection, which may cause the model to lean toward negative class discrimination. However, identifying potential positive samples is more valuable in our objectives. Therefore, we opt for a pan-cancer analysis to address this issue. Our study employs the same approach as the EMOGI method to extract multi-omics features of genes. We utilize clinical data from TCGA to calculate the mutation rate, methylation value, and gene expression data for each gene, resulting in 48D initial input features. For detailed procedures, please refer to the Supplementary Material. To represent the interactions between genes, we use protein–protein interaction (PPI) data obtained from the CPDB (Kamburov *et al.* 2011) database and encode the relationships between proteins as gene–gene interactions, forming a foundational homogenous network. We only retain edges in the PPI network where the likelihood of the relationship is greater than 0.5 as the basis for gene–gene relationships. To better leverage rich prior biological knowledge and increase the model’s interpretability, we introduce an heterogeneous network by incorporating various multi-omics data related to cancer from the MSigDB (Liberzon *et al.* 2015) database as nodes, in addition to gene nodes. The specific types and quantities of nodes we add are listed in Table 1. We consider the relationships between these newly added nodes and genes, treating them as edges within the heterogeneous network. The number and types of edges in the network are presented in Table 2. Please refer to the diagram of the heterogeneous network structure we have provided in Supplementary Fig. S7. Our research utilize the latest version of the NCG database (NCG7.0) (Dressler *et al.* 2022) to define positive samples as cancer driver genes, including candidate oncogenes. We obtain a collection of potential cancer driver genes from the set of genes supported by literature or research evidence of the CancerMine database (Lever *et al.* 2019). The remaining genes after removing CancerMineDB collection are considered as negative samples. This process results in a dataset with 2226 positive samples and 4240 negative samples. Our method does not employ the approach of generating negative sample genes by excluding the direct neighbors of known cancer driver genes in the network. Instead, we use data mining to avoid introducing additional label information that could impact model performance. We filter negative samples using existing research on cancer driver genes, selecting genes as negative samples that have no research supporting their classification as potential cancer driver genes. Given that the sample labels represent approximately half of the entire gene pool, the model training process falls under the category of semi-supervised learning.

**Table 1.** Types and numbers of nodes in MCDHGN heterogeneous network.

Nodes types	No. of entities
Gene	12 944
Positional gene sets	293
Pathways	3089
MicroRNA	3708
Computational gene sets	858
GO	10 525
HPO	5404
Oncogenic signature	189
Cell type signature	829

**Table 2.** Relationships (edges) between nodes in MCDHGN heterogeneous networks.

Type	No. of entities
Gene–gene	314 032
Gene–positional gene sets	12 910
Gene-pathways	134 089
Gene-microRNA	596 322
Gene-computational gene sets	78 744
Gene-GO	701 315
Gene-HPO	385 926
Gene-oncogenic signature	24 034
Gene-cell type signature	112 393

### 2.2 Overview of MCDHGN

**Figure 1.**
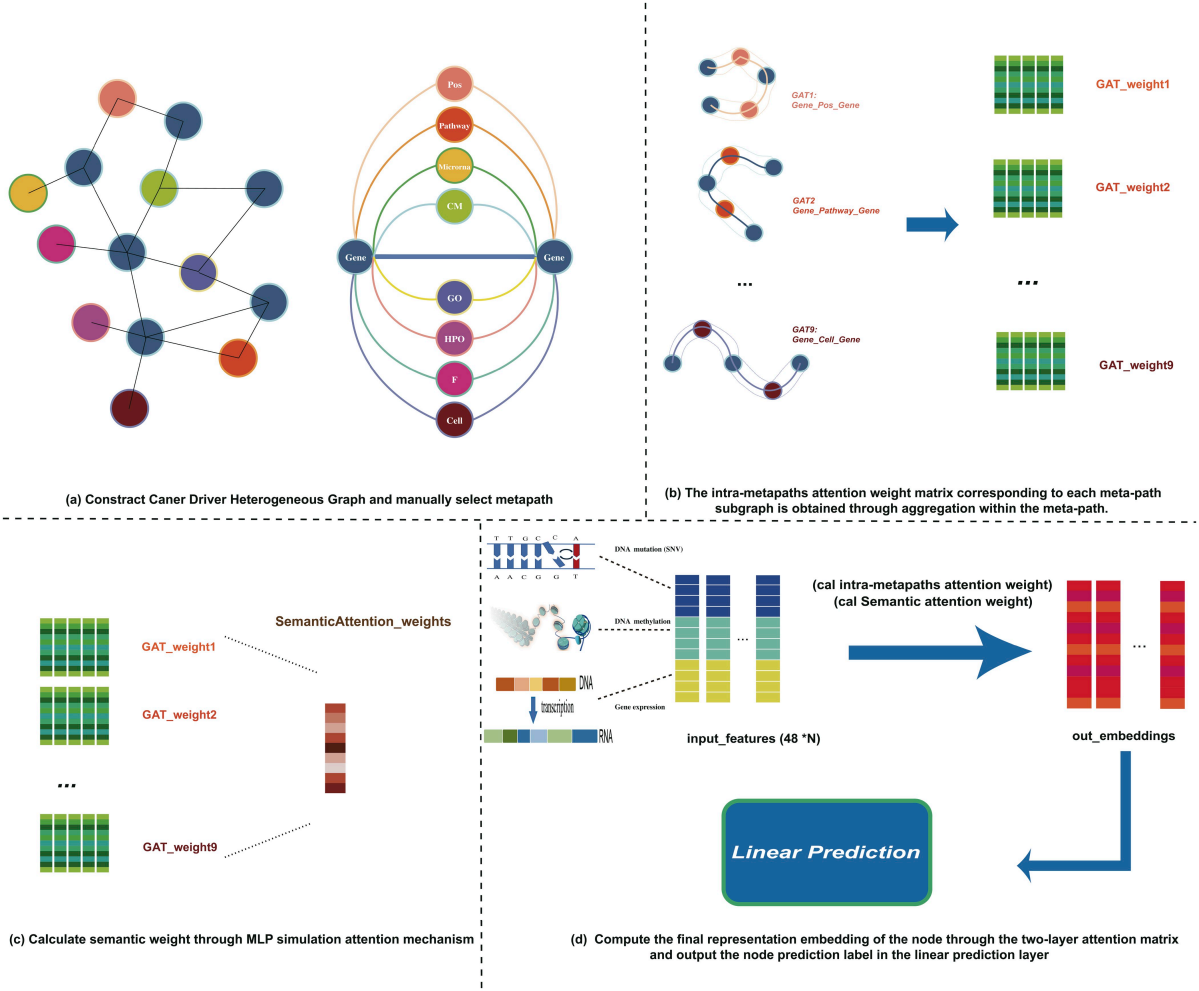
The framework of the MCDHGN model consists of four main components: First, we build a heterogeneous network of cancer driver genes and manually select meta-paths. Next, the model employs an attention mechanism to represent gene nodes through their neighboring nodes within specific meta-paths. Then, it aggregates the representations obtained from different meta-paths using attention weights. Finally, a linear classifier is used to complete the end-to-end output of the model’s predictions for cancer driver genes.

MCDHGN (Cancer Driver Gene Prediction using Multi-omics Heterogeneous Network and Meta-path Aggregation) consists of four main components: multi-omics heterogeneous network construction and manual selection of meta-paths; intra-meta-path information aggregation; inter-meta-path semantic weight aggregation; and the cancer prediction module based on a multi-layer linear classifier. The overall flowchart is illustrated in Fig. 1. Specifically, our model is an end-to-end architecture, starting with the input of nodes from the multi-omics heterogeneous network and the initial multi-omics features of each gene. The intra-meta-path information aggregation part resembles multiple parallel graph attention networks (GATs). It aims to learn the contribution weights of specific neighboring nodes within a certain meta-path for each gene node. On the other hand, the inter-meta-path semantic weight aggregation part employs a global attention mechanism to output the contributions of various meta-paths to the final prediction result. Finally, a multi-layer linear perceptron outputs a 2D vector representing the classification result. The model’s output is logarithmized and normalized, and the cross-entropy loss is then used to calculate the loss. Additionally, in the case analysis, the weights of the two parts in the meta-path aggregation module are output to explore the model’s interpretability.

### 2.3 Meta-path selection and meta-path-based MFGs extraction

We provide a basic definition of a heterogeneous network, where a HG is defined as a graph G={V,E}. For each node v∈V and each edge e∈E, they are connected through mapping relationships ϕ(v):V→A and φ(e):E→R, where *A* and *R*, respectively, represent all types of nodes and edges. We consider that the network pattern of graph *G* can be defined as S=(A,R), which can be understood as the graph defined on node types *A* with connections derived from *R*. A meta-path is based on the network pattern *S* and is represented as $m=A_{1}\overset{R_{1}}{\to }A_{2}\overset{R_{2}}{\to }\cdots \overset{R_{l}}{\to }A_{l+1}$, which can be abbreviated as $A_{1}A_{2}\cdots A_{l+1}$, where the node types $A_{1},A_{2},\ldots ,A_{l+1}\in A$. The types of edges $R_{1},R_{2},\ldots ,R_{l}\in R$. We establish a heterogeneous network that includes genes and multi-omics biological entities. When two gene nodes are connected through a certain biological entity, such as being associated with a specific biological process described as Pathway1, we create the meta-path gene_a-Pathway1-gene_b. During the process of information transmission, the information passed to gene_b includes not only the characteristics of gene_a but also captures the semantics of Pathway1. Thus, during interpretability analysis, we can use the semantics and biological interpretation of Pathway1, along with the biological functions of gene_a, to conduct an interpretable analysis of the impact this meta-path has on gene_b. For specific details on manually selecting meta-paths, please refer to the Supplementary Material. In the given meta-path Φ, we perform random walk sampling with limited steps based on neighbors along the meta-path to obtain a random walk sampling. During the process of random walks, the sampler selects the next node type strictly according to the meta-path specified. Starting from a particular node and following a meta-path of length *l*, we select *l* − 1 times to identify the sampling neighbors of the specific node based on this meta-path. The new graph of node relationships obtained by sampling a specific meta-path is referred to as the message flowing graph (MFG) of that meta-path. In this information transfer subgraph, the linkage between the initiating node and the destination node is determined by the intermediate nodes in the meta-path.

### 2.4 Internal message aggregation

In the information transfer subgraph, we use the attention mechanism between nodes to calculate the attention distribution among neighbor nodes of a certain node. For gene node *i*, firstly, linearly transform the vector *h_i_* of node *i* to get $h_{i}^{\prime }=W_{\Phi }h_{i}$. This step is to map the initial features to a unified hidden layer dimension. First, calculate the similarity $e_{ij}=\sigma (a_{\Phi }^{T}[h_{i}^{\prime }||h_{j}^{\prime }])$ between node *i* and all other nodes in the information transfer subgraph. Then, use the softmax method to normalize the contribution of node *j* to node *i*, and the attention score $\mathrm{alph}a_{ij}$ can be obtained. The overall process, as shown in Equation (1), incorporates the consideration of node *i*’s weight through self-attention mechanism. In this context, *k* and *j* can be same as *i*. Which implies that within the message subgraph, nodes can establish self-connections through meta-paths. The biological significance of these self-connections is determined by the node’s initial multi-omics features and the intermediate nodes traversed in the meta-path. The meaning of $N_{i}{\;}^{\Phi }$ is the set of all neighbor nodes adjacent to the *i* node in the Φ class message flow subgraph:

(1) $$\alpha_{ij}^{\Phi }=\frac{\;\text{exp}\;\left(\sigma \left(a_{\Phi }^{T}\cdot \left[h_{i}^{\prime }||h_{j}^{\prime }\right]\right)\right)}{\sum_{k\in N_{i}^{\Phi }}\;\text{exp}\;\left(\sigma \left(a_{\Phi }^{T}\cdot \left[h_{i}^{\prime }||h_{k}^{\prime }\right]\right)\right)}$$

Indeed, the representation results of the nodes in the meta-path message subgraph Φ need to pass through a non-linear activation function, as shown in Equation (2). This activation function introduces non-linearity to the model and allows it to capture more complex relationships and patterns in the data:

(2) $$z_{i}^{\Phi }=F.\mathrm{elu}\left(\sum_{j\in N_{i}^{\Phi }}\alpha_{ij}^{\Phi }\cdot h_{j}^{\prime }\right)$$

To enhance the expressive and generalization capabilities of the model, we use a multi-head attention mechanism to enhance the information content and expressiveness of intermediate vectors. Specifically, we perform the previous process many times to get $z_{i}^{\Phi \prime },z_{i}^{\Phi \prime \prime }$…. The results of the multi-head attention computations are directly concatenated. In the subsequent hyperparameter selection experiments, we will discuss the number of attention heads used for the intra-path aggregation process. The individual outputs of each meta-path for a single node will be concatenated to obtain the node’s representation vector. Finally, by concatenating the representation vectors of all nodes together, we obtain a final output matrix of dimensions N·out_dims·attention_heads called $Z_{\Phi }$.

### 2.5 Global semantic attention computation

By utilizing information propagation subgraphs and intra-path aggregation, we can ultimately obtain feature vector representations for gene nodes under each type of meta-path relationship. To fully integrate these features, we adopt a global attention mechanism. This mechanism calculates distinct semantic weights for each meta-path to differentiate the importance level among different meta-paths. In other words, we calculate the attention score for each meta-path as $\beta_{\Phi_{0}},\beta_{\Phi_{1}},\ldots \beta_{\Phi_{M}}$, First, expand the output matrix $Z_{\Phi_{i}}$ corresponding to the *M* meta-paths obtained in the previous step into a 2D matrix (N·H),H=out_dims·attention_heads. Then concatenate into matrix Z=M·N·H. We use a two-layer MLP to calculate the semantic weights between meta-paths, as shown in Equation (3):

(3) $$\omega^{\Phi_{i}}=\frac{1}{N}\sum_{j=1}^{N}(W_{2}(\tanh(W_{1}Z_{j}^{\Phi_{i}}+b)))$$

where *W*_1_ is the weight of the first layer of MLP, which is used to extract the non-linear features of $Z^{\Phi_{i}}$. Layer *W*_2_ is used to simulate the similarity comparison vector *q^T^* of the simple attention layer. The final result is normalized by a softmax layer, and the semantic weight $\beta^{\Phi_{i}}$ between different meta-paths can be obtained, as shown in formula (4):

(4) $$\beta^{\Phi_{i}}=\frac{\;\text{exp}(\omega^{\Phi_{i}})}{\sum_{j=1}^{M}(\text{exp}(\omega^{\Phi_{j}}))}$$

Finally, we calculate β·Z to obtain the final representation vectors matrix *X* for all nodes. In order to predict the final score, a linear prediction layer composed of a double MLP is used to calculate the classification results of the nodes. We use the form of score to get the probability that the node is classified into positive and negative classes, respectively, use the log_softmax function to convert the score into a probability distribution. Subsequently, the final loss function is computed utilizing the cross-entropy loss. As shown in formulas (5) and (6):

(5) $$\hat{y}=\mathrm{relu}\left(X\cdot W_{3}^{T}+b_{3}\right)\cdot W_{4}^{T}+b_{4}$$

 

(6) $$\text{loss}=-\text{log}\;p(y|\theta )=-\sum_{j=1}^{2}y_{j}\;\text{log}\;\hat{y_{j}}$$

where *y* represents the true label of the sample (one-hot encoding), $\hat{y}$ represents the label probability distribution predicted by the model. This article uses the AdamW optimization algorithm for backpropagation, by introducing the form of weight attenuation, a regular term is added to the loss, in order to manage the model’s complexity and mitigate the risk of overfitting. The final loss is shown by formula (7), where *ω_i_* is the parameter of the model, which can make the larger parameter suffer a larger penalty in the training, so that the parameter tends to a smaller value:

(7) $$\mathrm{Loss}=\mathrm{loss}+\lambda \cdot \sum_{i}\omega_{i}^{2}$$

## 3. Results

### 3.1 Experiment

We have selected eight baseline methods based on heterogeneous and homogeneous networks, which include both advanced network approaches in this field and both classic and advanced heterogeneous network methods. For specific details, please refer to the Supplementary Material. To facilitate comparison with state-of-the-art models, besides evaluating on the dataset built in this article, we also conducted experiments on the labeled dataset generated by the EMOGI method. We get the comparison of MCDHGN’s ability to classify cancer driver genes with eight baseline methods on a training set of two datasets. Each method uses the corresponding model constructed in the respective homogeneous or heterogeneous graph for experimentation. The evaluation metrics used are AUC (area under the ROC curve) and AUPR (area under the precision–recall curve). For parameter sensitivity analyses and operating environments, see Supplementary Fig. S2, and the section “Evaluation index and parameter sensitivity analysis” in Supplementary Material.

**Figure 2.**
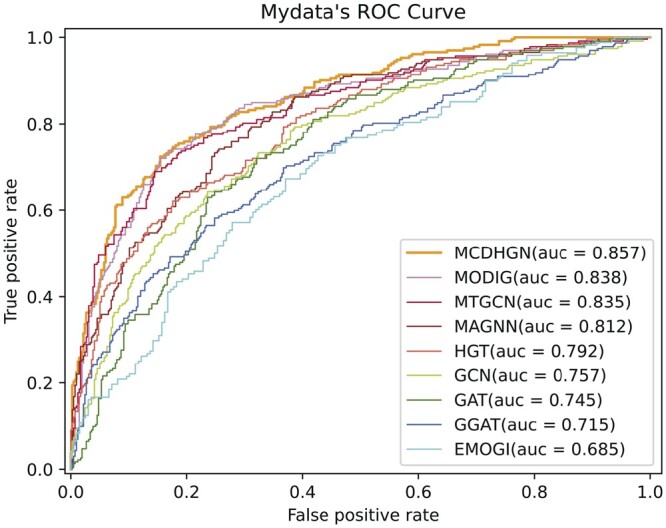
The performance of each method on our label dataset in terms of ROC curves.

**Figure 3.**
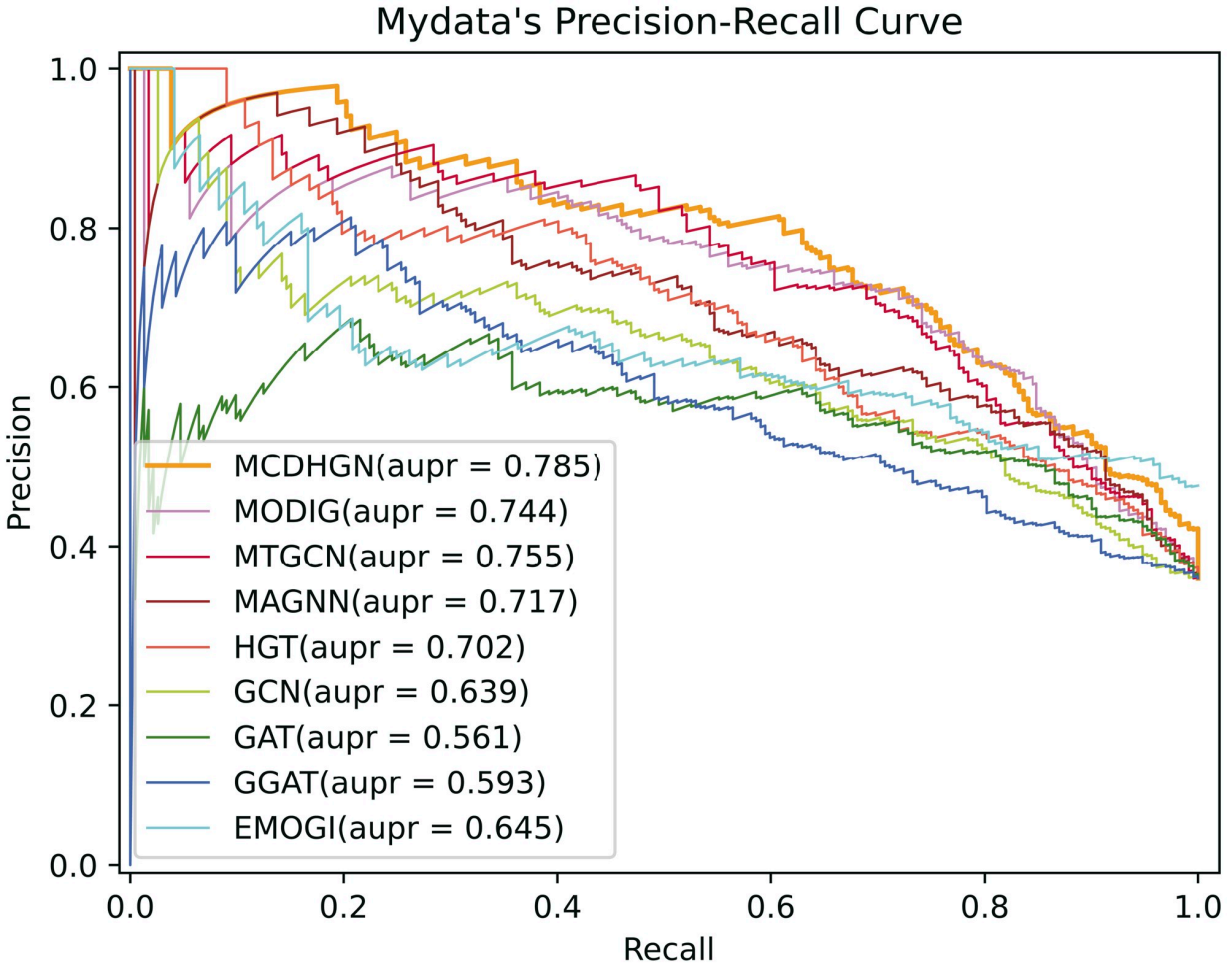
The performance of each method on our labeled dataset in terms of precision–recall (P–R) curves.

From Table 3, it can be observed that our model performs optimally in all evaluation metrics. The smaller number of positive samples in the EMOGI dataset, without considering NCG’s candidate cancer genes, strengthens the correlation between positive samples, making the feature patterns more evident. Additionally, during the generation of negative samples in the EMOGI dataset, the potential negative sample genes directly connected to known cancer driver genes in the PPI network are removed, making the feature patterns of negative samples more apparent. In contrast, our dataset considers a broader range of genes, and the generation of positive and negative samples relies solely on literature data mining without incorporating additional prior knowledge. This leads to better performance in predicting potential cancer driver genes. Moreover, it can be observed that heterogeneous network models overall outperform homogeneous network models, and heterogeneous network models provide more significant room for interpretability. These observations collectively demonstrate the superior performance of our proposed MCDHGN model in predicting cancer driver genes, and the benefits of using heterogeneous networks for enhanced performance and interpretability. Figures 2 and 3 demonstrate the prediction performance of MCDHGN with the new labeled dataset, and Supplementary Fig. S3 shows its performance on the emogi dataset, it can be noticed that MCDHGN demonstrates better performance than the baseline method on the test set of both two datasets, showing strong generalization capabilities. The model’s ability to generalize well to unseen data is an important aspect of its effectiveness and reliability. Furthermore, the semantic weighting module’s output of the nine meta-paths’ weight proportions, which represent their contributions to the final prediction, is shown Supplementary Fig. 4. The figure illustrates significant differences in the contributions of different meta-paths. The MCDHGN model effectively identifies crucial semantic modules, allowing us to pinpoint relevant biological justifications more easily. The ability to identify and emphasize important semantic modules provides the MCDHGN model with an advantage in capturing biologically relevant information and contributing to its superior predictive performance.

**Table 3.** Results of classification performance on the validation set.

Methods	Mydata AUC	Mydata AUPR	EMOGI data AUC	EMOGI data AUPR
GCN (Kipf and Welling 2016)	0.7744 ± 0.0129	0.6842 ± 0.0090	0.8455 ± 0.0128	0.7179 ± 0.0290
GAT (Veličković *et al.* 2017)	0.7578 ± 0.0244	0.6355 ± 0.0359	0.8441 ± 0.0170	0.7004 ± 0.0280
GGAT (Qiu *et al.* 2021)	0.7264 ± 0.0149	0.6213 ± 0.0190	0.7678 ± 0.0130	0.6213 ± 0.0343
HGT (Hu *et al.* 2020)	0.8262 ± 0.0064	0.7421 ± 0.0191	0.8725 ± 0.0089	0.7565 ± 0.0204
EMOGI (Schulte-Sasse *et al.* 2021)	0.6680 ± 0.0171	0.5258±0.0142	0.8097±0.0083	0.6883 ± 0.0151
MTGCN (Peng *et al.* 2022)	0.8384 ± 0.0136	0.7750 ± 0.0112	0.8935 ± 0.0200	0.8112 ± 0.0293
MODIG (Zhao *et al.* 2022)	0.8152 ± 0.0109	0.7421 ± 0.0117	0.8977 ± 0.0147	0.7922 ± 0.0124
MAGNN (Fu *et al.* 2020)	0.8272 ± 0.0084	0.7040 ± 0.0130	0.8902 ± 0.0109	0.7892 ± 0.0252
**MCDHGN (our method)**	**0.8716 **±** 0.0111**	**0.8025 **±** 0.0111**	**0.9066 **±** 0.0170**	**0.8265 **±** 0.0170**

Bold value means the highest value in the column.

### 3.2 Ablation experiment

Study of ablation experiments was done using masking or changing part of the model architecture or input vectors. Firstly, the semantic aggregation module was replaced with a simple averaging module, removing the influence of the semantic module. The results of the nine meta-paths were summed using a simple average and denoted as *MCDHGN_interf_*. Next, the attention mechanism within each meta-path was removed, and a simple Graphconv module was used instead of GAT for intra-path aggregation, denoted as *MCDHGN_intraf_*. Based on the previous analysis, it was found that four meta-paths contributed more than 10% to the results. Therefore, these four significantly important meta-paths were separately used to construct a heterogeneous network to explore their impact on the model’s performance, referred to as $\mathrm{MCDHG}N_{\mathrm{top}4}$. Finally, the influence of the initial multi-omics input on the results was considered. Random vectors of the same dimension (48 dimensions) were used as new inputs to the model after normalization. This experiment was denoted as *MCDHGN_randomfeat_*. The final experimental results are presented in Table 4. The experimental outcomes reveal that diminishing or substituting various modules within the model results in a reduction of AUC and AUPR values. Among the ablation experiments, the most significant impact on the results comes from removing the attention mechanism within each meta-path. This indicates that identifying important neighboring nodes in the meta-path message-passing subgraph is crucial for predicting cancer driver genes. Additionally, replacing the initial features with random vectors also results in a noticeable performance drop. This information directly reflects the abnormal expression and mutation phenomena in cancerous tissues. Given the complexity of the heterogeneous semantics used in our model, enhancing the relevance of the initial information to the prediction task can significantly improve the model’s generalization ability. This enhancement enables the node features to possess stronger representational power, aiding the model in better capturing the characteristics and patterns associated with cancer driver genes. Regarding the changes in semantic weights between meta-paths and using only the top four meta-paths with the highest weights, the impact on performance is not as evident as observed with the two previous factors (removing intra-path attention and replacing initial features). However, preserving more meta-paths and the semantic-level attention contributes to enhancing the model’s interpretability.

**Table 4.** Ablation experiment results.

Methods	Five folds AUC	Five folds AUPR
MCDHGN	0.8716 ± 0.0111	0.8025 ± 0.0111
${\text{MCDHGN}}_{\mathrm{interf}}$	0.8591 ± 0.0071	0.7768 ± 0.0155
${\text{MCDHGN}}_{\mathrm{intraf}}$	0.8109 ± 0.0046	0.6906 ± 0.0124
${\text{MCDHGN}}_{\mathrm{top}4}$	0.8521 ± 0.0296	0.7642 ± 0.0540
${\text{MCDHGN}}_{\mathrm{randomfeat}}$	0.8121 ± 0.0072	0.7208 ± 0.0194

### 3.3 Results

**Figure 4.**
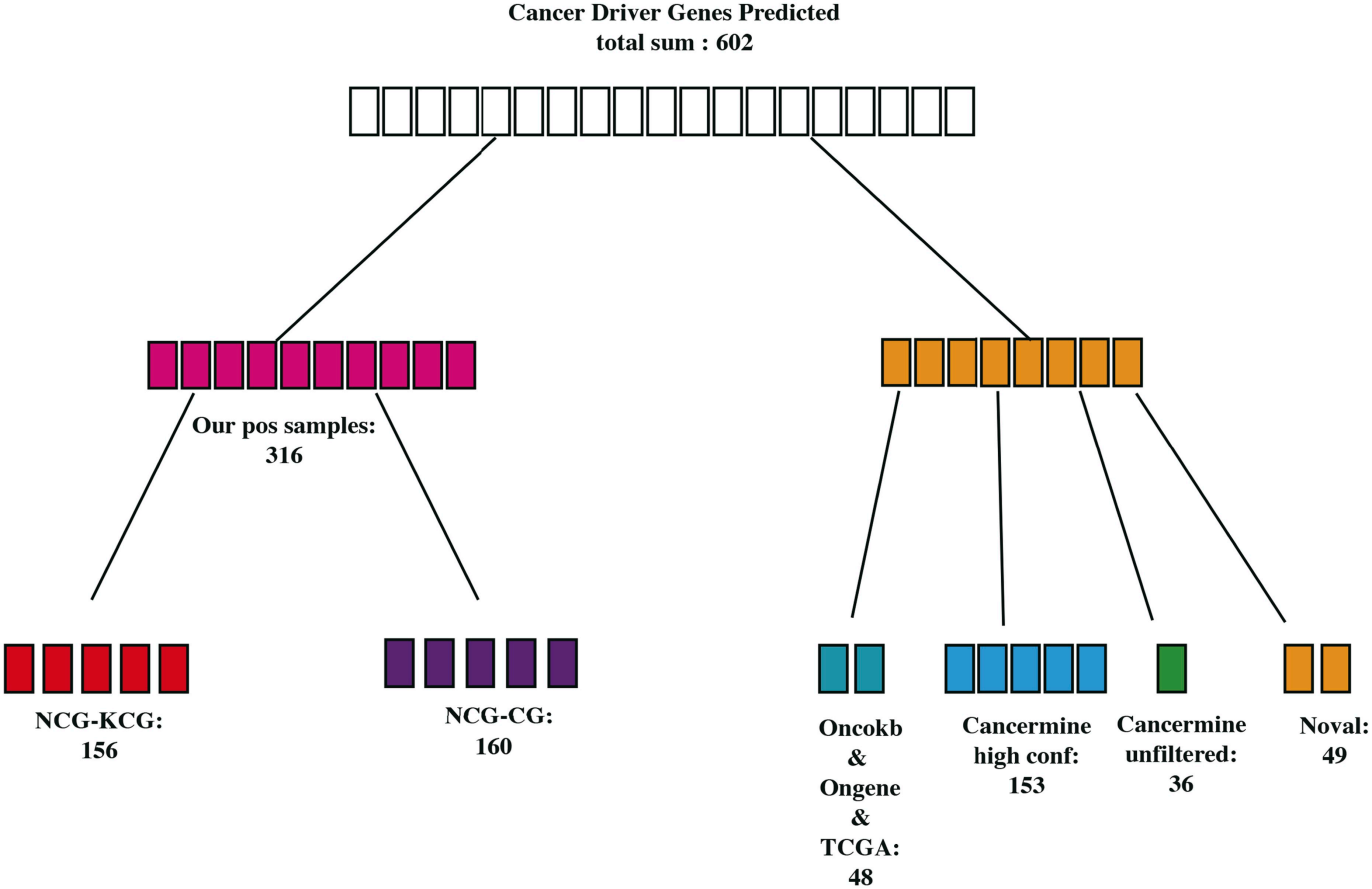
Suspected cancer driver genes predicted by MCDHGN.

In the cancer driver gene prediction phase, we used the entire training dataset for training and then outputted the prediction scores for all gene nodes. We considered samples with scores greater than 0.8 as potential cancer driver gene predictions, resulting in 602 suspected cancer driver genes (as shown in Fig. 4). Analysing the 602 suspected cancer driver genes, we found that 323 genes were already present in the positive sample set, specifically in the NCG7.0 database. Among them, 156 genes were known cancer driver genes (KCGs), and 160 genes were candidate cancer driver genes (CGs) according to NCG set. Then, we continued our analysis on the remaining 254 genes. Among these, 48 genes were mentioned in the Oncokb database (Chakravarty *et al.* 2017), Ongene database, and a comprehensive cancer analysis paper from TCGA (Ding *et al.* 2018), indicating some evidence of their involvement in cancer. Using the Cancer Miner data mining tool, they further explored potential cancer genes. Cancer Miner identified 153 genes as high-confidence cancer driver genes and 36 unfiltered genes were mined for which some studies have shown that these genes are associated with cancer but no direct evidence. In summary, 91.86% of cancer driver genes classified by MCDHGN have at least one paper or database evidence mentioning them as cancer driver genes or cancer-related genes. The remaining 49 genes (8.14%) are predicted as novel cancer driver genes that require further validation since there is currently no direct research evidence supporting their role in cancer.

### 3.4 Case study

We will illustrate how MCDHGN demonstrates biological interpretability in predicting cancer driver genes by presenting case studies on the SETD2 and ZBTB17 genes.

From Table 5, it is evident that the non-positive sample gene ZBTB17 is most strongly associated with the cancer driver gene PTEN. Both ZBTB17 and PTEN are part of the WP_SMALL_CELL_LUNG_CANCER pathway, indicating their relevance to small cell lung cancer. The PTEN protein primarily functions by regulating the PI3K/Akt signaling pathway. When PTEN function is impaired, excessive activation of the PI3K/Akt signaling pathway may lead to abnormal cell proliferation and survival, promoting tumor development. It can be inferred that ZBTB17 is also involved in the regulation of cell signaling pathways, as research (Ikegaki *et al.* 2007) suggests its association with tumor occurrence and development. ZBTB17 interacts with the transcription factor Myc, influencing the expression of Myc target genes. Myc is an important cancer-related gene associated with tumor cell proliferation, survival, and metastasis. Both Myc and the PI3K/AKT signaling pathway can jointly promote tumor cell proliferation, survival, and invasion, leading to tumor progression. Hence, it is reasonable to believe that PTEN is a cancer driver gene that interacts or mutually influences ZBTB17 gene. Our model can determine a gene as a cancer driver gene based on the neighbors of specific genes in the heterogeneous network. It can also discuss the functional similarity of potential oncogenes to known oncogenes based on the biological annotations of the intermediate nodes of the meta-paths connecting different genes. By identifying the more influential meta-pathways and their intermediate nodes, the interpretability of the model can be improved. Please refer to the Supplementary Material for the case analysis of the SETD2 gene.

**Table 5.** Heterogeneous graph nodes and paths judging the top 10 contributions of ZBTB17 as a cancer driver gene.

Order	Genename	Meta-path type	Relevant biological explanations
No. 1	PTEN	Gene–CM–Gene	The primary function of the PTEN (Álvarez-Garcia *et al.* 2019) protein involves the regulation of the PI3K/Akt signaling pathway, PTEN and the target gene belong to CM1: MORF_MT4 CM2: MORF_PSMF1
No. 2	TP53	Gene–Pathways–Gene	The mutation of TP53 gene is closely related to the occurrence and development of various cancers (Petitjean *et al.* 2007)
No. 3	PTEN	Gene–Pathways–Gene	Both belongs to Pathway: WP_SMALL_CELL_LUNG_CANCER
No. 4	GRIK5	Gene–CM–Gene	Both belongs to CM1: MORF_MT4 CM2: MORF_PSMF1
No. 5	RUNX1	Gene–CM–Gene	Both belongs to CM1: MORF_MT4 CM2: MORF_PSMF1
No. 6	PGR	Gene–Gene	In breast cancer, PGR also plays an important role
No. 7	ESR1	Gene–Gene	The research (Gu and Fuqua 2016) shows ESR1 gene encodes estrogen receptor*α* which related to the occurrence of breast cancer
No. 8	SIM1	Gene–F–Gene	Observing the functional compensation phenomenon of RB1 and P130 genes In the experiment, after knocking out the P130 gene, both expression levels increased in the process of cell mitosis.
No. 9	TRIM63	Gene–Gene	(Peris-Moreno *et al.* 2020) shows TRIM63 plays an important regulatory role in the process of muscle atrophy
No. 10	PAX9	Gene–CM–Gene	Both belongs to CM1: MORF_MT4 CM2: MORF_PSMF1

## 4. Conclusion

In this study, we introduce a cancer driver gene prediction model called MCDHGN, which is based on heterogeneous network meta-path analysis. We employ random walks to generate informative subgraphs for each meta-path and aggregate information within and between meta-paths to produce the final gene representation vectors. We compare MCDHGN against state-of-the-art methods on two cancer driver gene label datasets and achieve superior performance. Subsequently, we use the output from MCDHGN for predicting cancer driver genes and attempt to provide some interpretable analysis of our prediction results. In our subsequent work, we plan to enhance the network’s expressive capabilities by using methods that augment the semi-supervised label dataset. We also consider incorporating the model’s topological features within the network to strengthen the initial multi-omics features. Once the model’s representational and generalization capabilities are well-developed, we will initiate efforts toward predicting specific types of cancer driver genes with small positive sample labels.

## Supplementary Material

btae362_Supplementary_Data
