# Supplementary material for: MCDHGN: heterogeneous network-based cancer driver gene prediction and interpretability analysis
Source: Bioinformatics. 2024 Jun 12;40(6):btae362. doi: 10.1093/bioinformatics/btae362 (PMC11256962; doi:10.1093/bioinformatics/btae362)
Supplement: btae362_Supplementary_Data [file btae362_supplementary_data.pdf]

Supplemental materials

Introduction

We did the following works in this paper:

(1) Extraction of initial features of gene nodes and construction of heterogeneous networks. A 48-dimensional multi-omics vector is generated for each gene based on the mutation probability of the gene in the tumour cells of the GEO clinical cases, including the probability of copy number variation (CNV) and single nucleotide variation (SNV); the probability of the occurrence of DNA methylation; and the expression product level of the gene. The heterogeneous network is also constructed in conjunction with gene expression product interrelationships and knowledge of relevant biological pathway annotations in preparation for subsequent tasks.

(2) Metapath-based Feature Extraction for Heterogeneous Networks and Cancer-Driven Gene Prediction Based on Heterogeneous Networks Node Classification, designs custom metapaths for message aggregation in heterogeneous networks, samples different kinds of metapaths in heterogeneous networks using neighbourhood-based random wandering sampling. And using the attention mechanism to aggregate messages within and between different meta-paths to get the final node embedding representation and to predict node attributes using multilayer perceptron (MLP).

(3) For the classification results of cancer driver genes, we compare the classification effectiveness of our model with current state-of-the-art methods and multiple GNN models, design ablation experiments to explore the factors affecting the model performance, make predictions of potential cancer driver genes using the full sample, present the prediction results and select two cases with high confidence from them to conduct a demonstration line case study, and find supportive dissertation evidence for the analysis results.

Initial features

The initial features of the genes are composed of three parts, resulting in a final vector dimension of 48*N, where N is the number of gene nodes. For detailed feature extraction scripts and code, please refer to https://github.com/1160300611/MCDHGN/tree/main/preprocess_data.

The specific three components are:

Gene Mutation Frequency: We calculate the gene mutation frequency using the occurrence rate of single nucleotide variations (SNVs) from the MAF (Mutation Annotation Format) files of patients in the TCGA database. MAF files contain detailed information about single nucleotide variations, which are typically produced by cancer genome projects like TCGA (The Cancer Genome Atlas). Using GENCODE annotations, our scripts are able to compute the normalized SNV frequency based on the length of the exon genes. When preprocessing MAF files for individual cancer types, the first step is to load the MAF file and remove non-silent mutations. Next, hypermutated samples are eliminated based on a list of hypermutated samples. Subsequently, a gene × sample matrix is calculated. Finally, if necessary, the matrix is normalized based on gene length. The averages of all gene-sample matrices for 16 types of cancer are calculated, resulting in an average matrix with dimensions of 16*N. This matrix represents the average SNV mutation frequency of each gene in TCGA samples for each type of cancer.

Gene Methylation: DNA methylation data for tumor and normal samples from the 450k Illumina bead array are processed using the get_mean_sample_meth.py script to compute the methylation matrices for both tumor and normal samples. This script also defines the promoter regions of genes and assigns each measured CpG site along with its distance from the transcription start site (TSS) to a specific gene. By calculating, the average methylation matrices for tumor and normal samples are obtained, and differential DNA methylation is calculated using log2 fold changes.

$$\mathrm{ratio}_f\mathrm{c} = \log_2\left(\frac{tumor_{samples}}{mean(normal_{samples})}\right) \qquad \text{equation 1}$$

$\mathrm{tumor}_{\mathrm{samples}}$ is the heterogeneity value of tumor samples. $\mathrm{mean}(\mathrm{normal}_{\mathrm{samples}})$is the average heterogeneity value of normal samples. Ultimately, we obtain a 16*N matrix representing the differential DNA methylation expression for 16 different types of cancer.

Gene expression: preprocess_gene_expression.py is a script for preprocessing gene expression data, which has been quantified using the FPKM method (Fragments Per Kilobase of transcript per Million mapped reads), including normal and tumor tissue samples from TCGA (The Cancer Genome Atlas), as well as normal tissue samples from GTEX. In the application of this script, only normal tissue data from GTEX and tumor tissue data from TCGA are used to calculate the log2 fold changes between them. This method is commonly used to compare gene expression levels in

different samples, such as normal and pathological tissues, to identify differentially expressed genes that may play a crucial role in the development of diseases. The processing workflow involves reading the gene expression data for tumor and normal tissues, ensuring that the genes (rows) in both datasets are aligned, calculating the ratio of the median expression of each gene across all tumor samples to the median expression across all normal samples, and taking the log base 2 of these ratios to obtain the log2 fold changes, as shown in equation 2.

$$FC_c = \log_2\left(\frac{\text{median}(P_c)}{\text{median}(N_t)}\right) \qquad \text{equation 2}$$

Ultimately, a 16*N matrix is obtained, representing the log2 fold changes of N different genes across 16 types of cancer samples compared to normal tissue samples, reflecting the differential expression of genes between cancerous and normal tissues.

Datasets

CPDB is an online cancer cell line proteomics database that encompasses protein expression data derived from large-scale mass spectrometry and RNA sequencing techniques for thousands of cancer cell lines. It serves as a valuable resource for cancer treatment and research. For our study, we specifically utilized the protein-protein interaction (PPI) network data available in CPDB. This network represents the relationships between genes based on the interactions between their protein products. The Human MsigDB collection is an online repository designed for the analysis of genomic data. It consists of various specialized gene sets that can identify gene expression patterns related to different biological processes, diseases, and drug responses. The MsigDB collection is composed of two main components: Clinically validated signal pathway collections and gene or protein annotation information from clinical trials. These biological process and entity annotations contribute to a better understanding of the interactions between pairs of genes and the linkages under common belonging to the same physiological process; Computationally derived gene sets that provide highly informative summaries of gene information in specific contexts. These gene sets are generated using modern high-throughput sequencing technologies and computational methods. For all nine types of nodes, we only consider the intersection with the genes present in the CPDB network to construct the heterogeneous network. Additionally, we manually define nine types of meta-paths. Utilizing meta-paths allows us to fully leverage the diverse types of nodes and edges present in the heterogeneous network, thereby creating a new way to describe gene interactions more effectively and comprehensively. Selecting appropriate meta-paths helps to better reflect the relationships between genes while also carrying some biological significance. This approach allows us to strike a balance between the predictive performance and interpretability of the model.

Extracted meta-paths:

In Supplementary Figure 7, we provided a diagram of the heterogeneous network. The biological entity descriptions before the nodes can be matched one-to-one with Table 2-1 in the main text. The content in parentheses represents the abbreviated form of intermediate nodes within the meta-path, as shown in Supplementary Figure 1.

The final extracted meta-paths, along with their biological nodes and the semantics of the paths, are presented in Supplementary Table 1. The abbreviated names within parentheses for intermediate nodes correspond to the node names shown in Supplementary Figure 1. Additionally, different colors of edges in Supplementary Figure 1 represent different types of meta-path connection patterns.

Baseline Introduction:

GCN (Kipf and Welling, 2016):This method is a commonly used network convolution algorithm for processing homogeneous graph data. Here, a three-layer GCN convolution approach is employed for computation in the homogeneous network.

GAT (Veliˇckovi´c et al., 2017):This method implementation uses the attention mechanism to compute the representation of a node on a neighbouring node using masked attention

GGAT (Qiu et al., 2021):This method introduces a gated network to learn the weights of each attention head, allowing for dynamic adjustment of attention head weights.

HGT (Hu et al., 2020):An attention model for heterogeneous networks, where nodes are considered as entities and edges as relations. Entity vectors are learnable representation vectors, eliminating the need for entity vector initialization and using random vectors instead.

EMOGI (Schulte-Sasse et al., 2021):This method is based on GCN and uses multiple omics data such as genomics and gene expression data as gene features to predict pan-cancer driver genes in a PPi network.

MTGCN (Peng et al., 2022):Building upon the EMOGI method, this approach considers situations where nodes in the homogeneous network have no interactions with each other. It adds a multi-task module for link prediction to enhance the model’s learning of input samples.

MODIG (Zhao et al., 2022):This method takes into account multi-dimensional homogeneous networks, where gene-gene interaction networks are generated based on multiple common relationships. It employs a joint learning mechanism based on GAT in multi-dimensional homogeneous networks.

MAGNN (Fu et al., 2020):This method is a commonly used meta-path-based approach for heterogeneous networks. Considering the limitations of the computational scale, it is not possible to perform full graph operations on the entire heterogeneous map, only Mini-Batch learning methods are feasible for training.

Evaluation index and parameter sensitivity analysis

Our research conducts parameter sensitivity experiments on a new dataset based on NCG7.0. First, the dataset is divided into a 9:1 ratio for the training and testing sets. The testing set is used to evaluate the model's generalization and prediction capabilities. The training set is further split into five folds for cross-validation, allowing for hyperparameter selection and evaluation during the five-fold cross-validation process. This three-set division aims to better utilize the data, prevent overfitting, and enhance the model's capacity for generalization. Subsequently, the model will be compared with baseline methods on both our dataset and the EMOGI dataset. The results on the validation set are the average values from the five-fold cross-validation. To assess the model's performance, this paper employs metrics such as the area under the ROC curve (AUC) and the area under the precision-recall curve (AUPR). For hyperparameter selection, a greedy strategy is employed, testing a set of hyperparameters and then fixing the best-performing ones for subsequent experiments. Four groups of hyperparameters are tested: the learning rate for the AdamW optimization algorithm {0.001, 0.005, 0.01, 0.015}, the number of attention heads affecting the internal aggregation output dimension of meta-paths {1, 2, 4, 8}, the dropout rate for attention weights {0.3, 0.4, 0.5}, which is a regularization technique applied in the attention mechanism to control the dropout rate and enhance model robustness, prevent overfitting, and improve generalization ability, and the hidden layer dimension within GAT (Graph Attention Network) during meta-path internal aggregation {128, 256, 512}, which impacts the output dimension of the meta-path aggregation. The model's architecture is inspired by the HAN (Heterogeneous Attention Network) model, and the meta-path internal aggregation part is implemented using the DGL (Deep Graph Library) framework. The overall code is implemented in Python 3.9 with Torch 1.12+ CUDA 10.2 architecture. The final selected hyperparameters are 0.01 learning rate, 4 attention heads, 0.4 attention weight dropout rate, and a hidden layer dimension of 256. These choices are made based on a combination of results and computational performance considerations.

Case study

Taking the SETD2 gene as an example, it is a known cancer driver gene in the NCG database. The paper utilized the contribution weight matrix matrix α and the semantic-level attention weight matrix β, calculated from the model, and multiplied them to obtain the specific contributions of each node within the metapaths to the SETD2 gene. Then, the top 10 gene nodes contributing to SETD2 and the corresponding metapaths were selected. The thickness of the lines in Supplementary Figures 1 represents the magnitude of their contributions. The detailed mechanism of their effects is shown in Supplementary Table 5. From Supplementary Figures 1, we can observe that the three metapaths shown in the figure correspond to the three metapaths with the highest weights, as calculated in the analysis. The gene-gene relationship corresponds to the PPi network from the CPDB database. The Gene-Pathways-Gene metapath includes Pathways nodes from the KEGG and Reactome Pathway databases. The Gene-CM-Gene metapath involves CM (Consensus Module) nodes derived from a study based on DNA microarray tumor expression data, which conducted a comprehensive analysis of microarrays from 22 tumor types and described the expression profiles of different tumors through modules.

The purpose of this optimization is to use the idea of connection prediction to make the connection prediction results in our optimized initial vector initial PPI network closer to the real result, thereby increasing the prior information of the initial vector to improve the final classification result. Furthermore, during the process of metapath aggregation, we will experiment with different activation functions to observe their impact on the representation vectors. Additionally, we will explore the possibility of updating the representation vectors by combining the original feature values with the newly generated vectors. This fusion approach aims to preserve the essential characteristics of the original information, resulting in more informative and meaningful representations. In our future work, we will focus on refining the network architecture and optimizing the input initial vectors. This involves seeking better ways to utilize the available prior information to improve the overall performance of our model. By making these improvements, we aim to enhance the accuracy and efficiency of the MCDHGN model and ultimately contribute to the advancement of cancer driver gene prediction and analysis in multi-omics data.

From Supplementary Table 2 and Supplementary Figures 6, we can visually observe that the model ranks evidence supporting SETD2 as a cancer driver gene among the top entries. Additionally, it can be deduced that the SETD2 gene plays a crucial role in regulating gene expression and participating in extracellular protein modifications. Mutations or deletions in the SETD2 gene are often associated with abnormal histone methylation and gene expression, leading to dysregulation of cell growth, apoptosis, and differentiation. These findings align with the conclusions drawn from actual research (Li et al., 2016) .

Supplementary Figures 1: Different types of Meta-paths of MCDHGN Network Different colors represent different types of nodes.

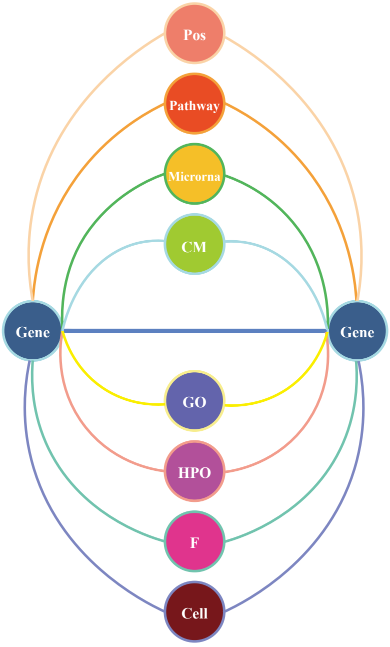

Supplementary Figures 2: Parameter sensitivity analysis results

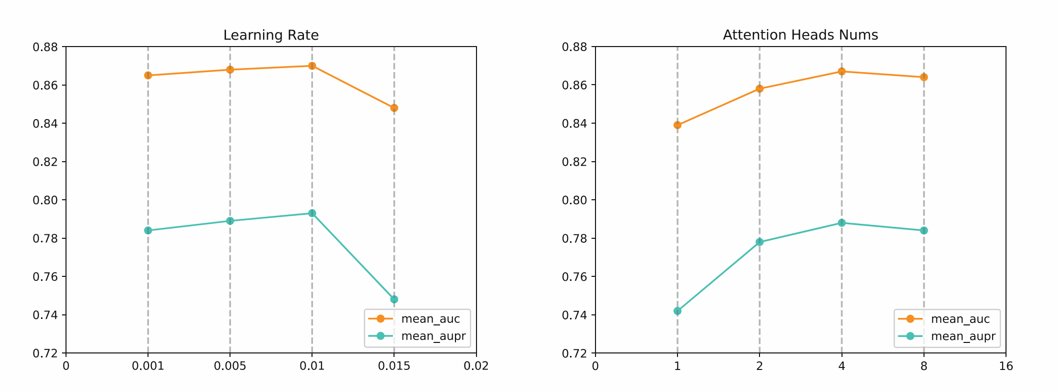

(a) adamW learning rate

(b) attention heads num

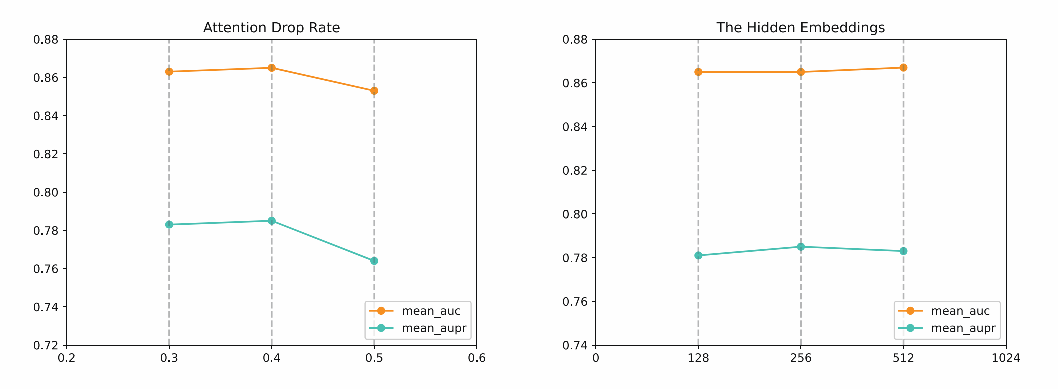

(c) attention drop rate (d) hidden embeddings num

Supplementary Figures 3: Comparative experimental results on the EMOGI test set

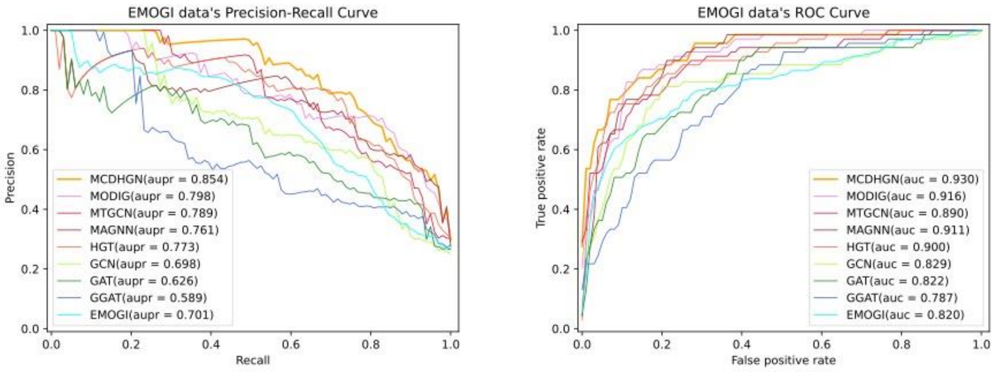

(a) EMOGI roc Curve (b) EMOGI PR Curve

Supplementary Figures 4: Meta-path semantic weight of each intermediate node in MCDHGN prediction results

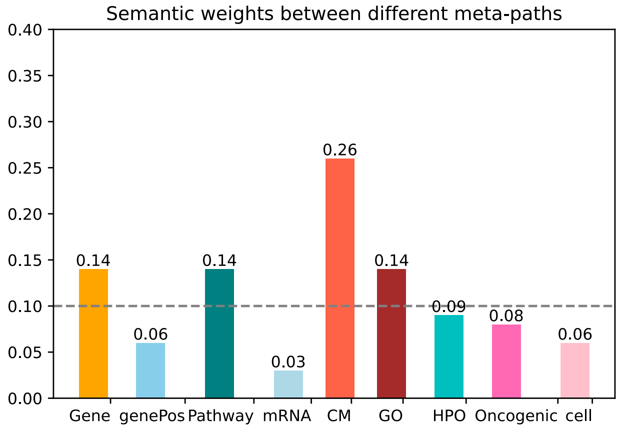

Supplementary Figures 5: Case analysis of ZBT17 gene

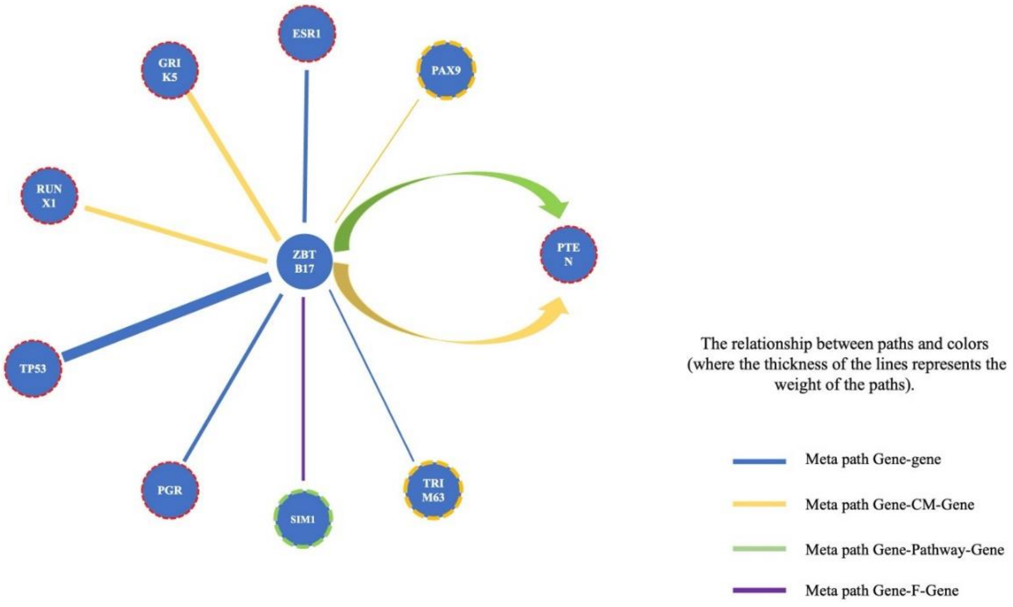

Supplementary Figures 6: Case analysis of SETD2 gene

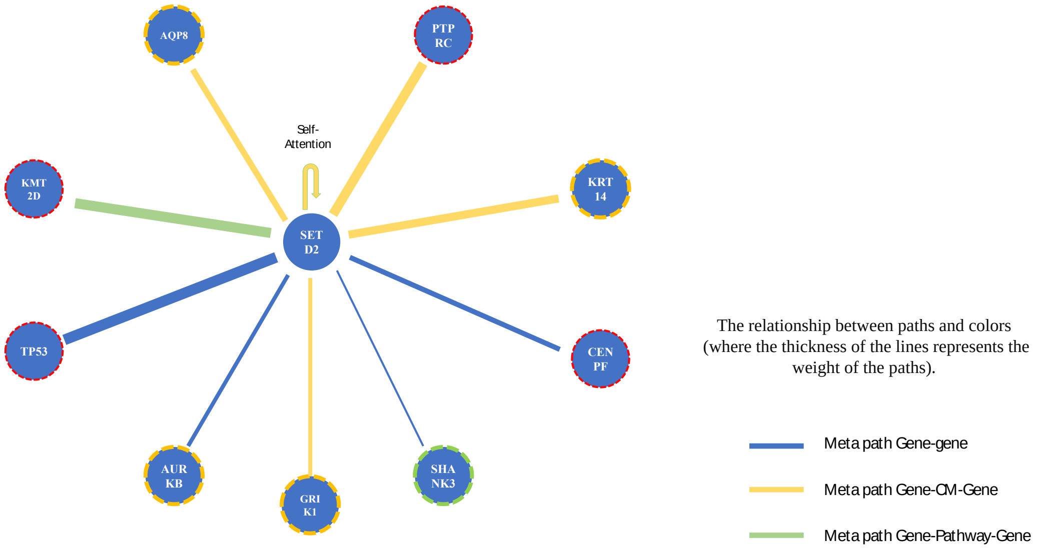

Supplementary Figures 7: Heterogeneous network with node name annotation

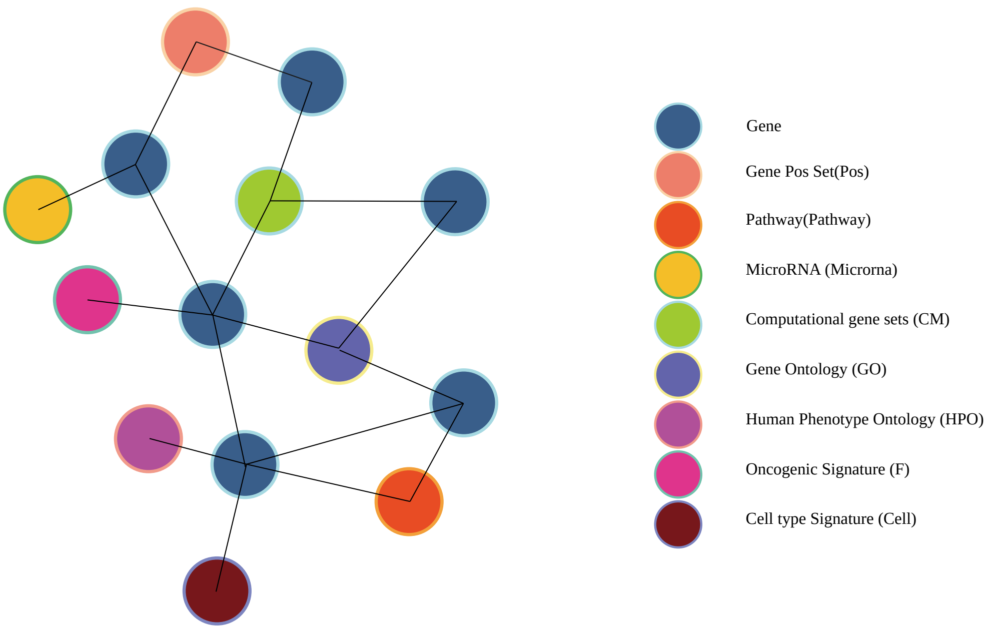

Supplementary Table 1: The nine manually extracted meta-paths and their edges' biological semantics in our study.

metapath src node	entity relationship	metapath intermediate node	entity relationship	Meta path dst node
Gene	Associate	None	Associate	Gene
Gene	Belong to	Gene Pos Sets (Pos)	Contains	Gene
Gene	Involved in	Pathways (Pathway)	Association to	Gene
Gene	Express	MicroRNA (Microrna)	Reverse transcription	Gene
Gene	Calculated	Computational gene sets (CM)	Calculated by	Gene
Gene	Comment	Gene Ontology (GO)	Describe	Gene
Gene	Comment	Human Phenotype Ontology (HPO)	Describe	Gene
Gene	Manifest	Oncogenic Signature (F)	Manifest in	Gene
Gene	Involved in	Cell type Signature (Cell)	Association to	Gene

Supplementary Table 2: Heterogeneity graph nodes and paths judging the top 10 contributions of SETD2 as a cancer driver gene.

Order	Gene name	Metapath type	Relevant biological explanation
No.1	TP53	Gene-Gene	TP53 gene is one of the important tumor suppressor genes in human. (Petitjean et al., 2007) p53 protein can regulate cell cycle, DNA repair, apoptosis, etc.
No.2	KMT2D	Gene-Pathways-Gene	Pathway1: REACTOME_CHROMATIN Chromatin modifying enzyme pathway; Pathway2: REACTOME_PKMTS Mechanism of methylation of histone lysine; Pathway3: WP Histone modification
No.3	PTPRC	Gene-CM-Gene	CM-126: Genes related to the role of DDX5: Studies (Nyamao et al., 2019) have shown that DDX5 gene is involved in the process of tumor cell proliferation, invasion and metastasis
No.4	KRT14	Gene-CM-Gene	Both belongs to CM computing module MODULE_94
No.5	AQP8	Gene-CM-Gene	Both belongs to CM computing module MODULE_94
No.6	CENPF	Gene-Gene	High expression of CENPF gene in cancer cells can promote the process of mitosis (Lin et al., 2016); This leads to chromosomal abnormalities and instability, which in turn promotes the occurrence and development of tumors

No.7	GRIK1	Gene-CM-Gene	Both belongs to CM computing module MODULE_94
No.8	AURKB	Gene-Gene	*AURKB is a gene encoding protein,* (Borah and Reddy, 2021) *which plays an important regulatory role in the process of cell mitosis.*
No.9	SETD2	Gene-CM-Gene	self-linkage of genes
No.10	SHANK3	Gene-Gene	There is currently no direct evidence linking this gene to cancer

Reference

Naheed Arfin Borah and Mamatha M Reddy. Aurora kinase b inhibition: a potential therapeutic strategy for cancer. Molecules, 26(7):1981, 2021.

Achatz Petitjean, MIW Achatz, AL Borresen-Dale, P Hainaut, and M Olivier. Tp53 mutations in human cancers: functional selection and impact on cancer prognosis and outcomes. Oncogene, 26(15):2157–2165, 2007.

Shih-Chieh Lin, Chung-Yang Kao, Hui-Ju Lee, Chad J Creighton, Michael M Ittmann, Shaw-Jenq Tsai, Sophia Y Tsai, and Ming-Jer Tsai. Dysregulation of mirnas-coup-tfii-foxm1-cenpf axis contributes to the metastasis of prostate cancer. Nature communications, 7(1):11418, 2016.

Rose Magoma Nyamao, Jing Wu, Li Yu, Xiao Xiao, and Feng- Min Zhang. Roles of ddx5 in the tumorigenesis, proliferation, differentiation, metastasis and pathway regulation of human malignancies. Biochimica et Biophysica Acta (BBA)-Reviews on Cancer, 1871(1):85–98, 2019.

Thomas N Kipf and Max Welling. Semi-supervised classification with graph convolutional networks. arXiv preprint

arXiv:1609.02907, 2016.

PetarVeliˇckovi´c,GuillemCucurull,ArantxaCasanova,Adriana Romero, Pietro Lio, and Yoshua Bengio. Graph attention networks. arXiv preprint arXiv:1710.10903, 2017.

Xinyu Fu, Jiani Zhang, Ziqiao Meng, and Irwin King. Magnn: Metapath aggregated graph neural network for heterogeneous graph embedding. In Proceedings of The Web Conference 2020, pages 2331–2341, 2020.

Wei Peng, Qi Tang, Wei Dai, and Tielin Chen. Improving cancer driver gene identification using multi-task learning on graph convolutional network. Briefings in Bioinformatics, 23 (1):bbab432, 2022.

Ziniu Hu, Yuxiao Dong, Kuansan Wang, and Yizhou Sun. Heterogeneous graph transformer. In Proceedings of the web conference 2020, pages 2704–2710, 2020

Linling Qiu, Han Li, Meihong Wang, and Xiaoli Wang. Gated graph attention network for cancer prediction. Sensors, 21(6): 1938, 2021.

Wenyi Zhao, Xun Gu, Shuqing Chen, Jian Wu, and Zhan Zhou. Modig: integrating multi-omics and multi-dimensional gene network for cancer driver gene identification based on graph attention network model. Bioinformatics, 38(21):4901–4907, 2022.

Roman Schulte-Sasse, Stefan Budach, Denes Hnisz, and Annalisa Marsico. Integration of multiomics data with graph convolutional networks to identify new cancer genes and their associated molecular mechanisms. Nature Machine Intelligence, 3(6):513–526, 2021.

Jun Li, Gerben Duns, Helga Westers, Rolf Sijmons, Anke van den Berg, and Klaas Kok. Setd2: an epigenetic modifier with tumor suppressor functionality. Oncotarget, 7(31):50719, 2016.
